# Health Status, Quality of Life, Psychosocial Well-being, and Wearables Data of Patients With Active Ulcerative Colitis Receiving Filgotinib Therapy (FilgoColitis Study): Protocol for a Real-world Observational Study

**DOI:** 10.2196/42574

**Published:** 2023-05-08

**Authors:** Sandra Plachta-Danielzik, Lena Grasskemper, Karen Schmidt, Stefan Schreiber, Bernd Bokemeyer

**Affiliations:** 1 Competence Network IBD Kiel Germany; 2 Clinic of General Internal Medicine I University Hospital Schleswig-Holstein, Campus Kiel Kiel Germany; 3 Department Medicine I University Hospital Schleswig Holstein, Campus Kiel Kiel Germany

**Keywords:** ulcerative colitis, inflammatory bowel, filgotinib, wearable, eHealth, digital health, quality of life, fatigue, patient-reported outcome, real-world evidence, mobile phone

## Abstract

**Background:**

Filgotinib was approved in Germany for treating patients with moderate to severe active ulcerative colitis in November 2021. It represents a preferential Janus kinase 1 inhibitor. The FilgoColitis study began recruiting immediately after approval and aims to assess filgotinib effectiveness under real-world conditions with a particular focus on patient-reported outcomes (PROs). The novelty of the study design is the optional inclusion of 2 innovative wearables, which could provide a new layer of patient-derived data.

**Objective:**

The study investigates quality of life (QoL) and psychosocial well-being of patients with active ulcerative colitis during long-term exposure to filgotinib. PROs related to QoL and psychometric profiles (fatigue and depression) are collected alongside with disease activity symptom scores. We aim to evaluate physical activity patterns collected by wearables as an addition to traditional PROs, patient-reported health status, and QoL in different phases of disease activity.

**Methods:**

This is a prospective, single-arm, multicentric, noninterventional, observational study with a sample size of 250 patients. QoL is assessed with validated questionnaires: the Short Inflammatory Bowel Disease Questionnaire (sIBDQ) for the disease-specific QoL, the EQ-5D for the general QoL, and the fatigue questionnaire (Inflammatory Bowel Disease-Fatigue [IBD-F]). Physical activity data are collected from patients using wearables (SENS motion leg sensor [accelerometry] and smartwatch, GARMIN vívosmart 4).

**Results:**

The enrollment started in December 2021 and was still open at the date of submission. After 6 months of study initiation, 69 patients were enrolled. The study is expected to be completed in June 2026.

**Conclusions:**

Real-world data for novel drugs are important to assess effectiveness outside of highly selected populations represented by randomized controlled trials. We examine whether patients’ QoL and other PROs can be supplemented with physical activity patterns measured objectively. Use of wearables with newly defined outcomes represents an additional observational tool for monitoring disease activity in patients with inflammatory bowel disease.

**Trial Registration:**

German Clinical Trials Register DRKS00027327; https://drks.de/search/en/trial/DRKS00027327

**International Registered Report Identifier (IRRID):**

DERR1-10.2196/42574

## Introduction

### Background

Ulcerative colitis (UC) is a chronic disease, which affects the digestive system and therefore, belongs to inflammatory bowel diseases (IBDs). Worldwide, more than 1.8 billion patients have been diagnosed with UC [1] with an ever-increasing trend [2,3].

UC has a chronic recurrent course of disease. Patients with UC have recurrent diarrhea, abdominal pain, nausea, weakness, or anemia, among other symptoms. As a result, the patients’ quality of life (QoL) and well-being can be impaired [4,5].

Currently, there are already many targeted therapies for moderate to severe UC. The use of biologics and other small molecules in patients with UC is increasing. Nevertheless, effectiveness is only approximately 30%. Recently, new modes of action have been introduced. In 2019, tofacitinib became the first Janus kinase (JAK) inhibitor approved in UC. However, as a pan JAK inhibitor, it has elicited some serious adverse events. Due to several Dear Doctor Letters (Rote-Hand-Brief), its acceptance by physicians has declined. Filgotinib is the first oral preferential JAK1 inhibitor, with fewer side effects expected than with a pan JAK inhibitor, approved for UC in Europe as of November 2021. Because pivotal studies with narrow predefined inclusion criteria do not always reflect treatment in routine clinical practice, the FilgoColitis study was established to reflect use under real-world conditions.

The novelty of the study design is the optional inclusion of 2 innovative wearables, which could provide a new layer of patient-derived data. Correlations with disease activity and QoL have been established before [6-8], but the cohort sizes were small and not in a longitudinal setting around targeted therapy. Therefore, real-world studies to assess the effect of digital health tools on disease activity are still needed [9].

### Objectives

This study aims to investigate the QoL and psychosocial well-being of patients with UC on filgotinib therapy over the long-term course of different disease phases under routine conditions. Therefore, we aim to use wearables additionally and collect patient-reported outcomes (PROs) related to QoL and psychomotor profiles (fatigue and depression), including physical activity, under real-world conditions.

Using wearables, we aim to investigate whether physical activity data are related to QoL in patients with UC and whether there is an association between physical activity patterns, health status, and QoL, and additionally to define a new outcome in UC treatment. Furthermore, we will evaluate the safety profile of filgotinib therapy considering (serious) adverse events.

## Methods

### Study Design

The FilgoColitis study is a prospective, single-arm, multicentric, investigator-initiated, noninterventional observational study.

### Outcomes

#### Primary Outcome Measure

The disease-specific QoL of patients with UC during long-term course of therapy is the primary outcome and is assessed with the help of the validated and internationally widely applied Short Inflammatory Bowel Disease Questionnaire (sIBDQ, German version), which contains 4 domains: bowel symptoms, emotional health, systemic systems, and social function [10]. Thereby, the different disease phases will be considered and examined.

#### Secondary Outcome Measures

As a secondary outcome, the safety profile of the filgotinib treatment will be explored, taking (serious) adverse events into account. Furthermore, clinical parameters such as the disease activity using the partial Mayo Score (pMayo, and additionally PRO2) and the Montreal Classification are assessed. In addition, the therapy deemed necessary and useful for the patient by the physician is recorded. The general QoL using the validated questionnaire EQ-5D-5L (which essentially consists of 2 pages: the EQ-5D descriptive system and the EQ visual analog scale [EQ-VAS]) is documented. The descriptive system comprises 5 dimensions: mobility, self-care, usual activities, pain or discomfort, and anxiety or depression. Each dimension has 5 levels: no problems, slight problems, moderate problems, severe problems, and extreme problems [11].

Fatigue is highly prevalent among patients with IBD [12]; therefore, the fatigue questionnaire (Inflammatory Bowel Disease-Fatigue [IBD-F]) is used in the study [13]. The scale of the IBD-F questionnaire was developed to be used for the diagnosis of fatigue or monitoring fatigue levels over time. The German version of the questionnaire has been published recently [14].

#### Additional Exploratory Outcome Measures

Wearables will be given to patients to collect their physical activity data, such as the number of steps per day, pulse rate, calories burned per day, physical activity patterns (day and night), stress, sleep rhythm, and sleep quality. We aim to examine the correlation between physical activity, stress, sleep, disease phases, and the patients’ psychosocial impairment. The used wearables are (1) an accelerometry-based sensor (SENS motion, Class 1M medical device), which is attached to the thigh of one leg, and (2) a smartwatch (GARMIN vívosmart 4). The wearables are adapted for German server–based documentation of the physical activity parameters (number of steps per day and speed), pulse rate, calories per day, physical activity patterns (day and night), rhythm and quality of sleep, and stress level.

### Statistics

#### Overview

This study is descriptive, and no formal hypotheses are tested. Patient and clinical characteristics are described to capture the baseline situation of patients treated with filgotinib. Descriptive statistics are used to summarize patients’ demographics and baseline data. Specifically, for continuous variables, the mean, 95% CI for the mean, SD, median, and IQR are reported. For discrete variables, the frequency and number of missing observations are reported.

In this noncomparative observational study, the primary end point is analyzed as the mean change (the difference in scores between the 2 time points) in sIBDQ values from baseline to week 10 and is presented along with the 95% CI. For sIBDQ scores (total), mean changes from baseline to week 10 (week 52 and week 104) are calculated with a 1-sample *t* test for differences between paired observations (or a nonparametric analog).

Patients’ sIBDQ QoL is summarized and compared concerning the following: (1) mean change from baseline to assessment points during the study and (2) at each analysis time point, the number of patients who have completed the questionnaire is reported as a percentage of eligible patients. Data will be collected at visit 1 (ie, at baseline) and then at approximately week 10, and every 6 months up to 2 years.

#### Repeated Measurements Over Time

Each subject is assumed to have his or her mean response curve that explains how the sIBDQ score changes over time (intraindividual changes). A mixed effects model is used to model individual sIBDQ response curves over time.

#### Justification of Sample Size

The difference in sIBDQ scores from baseline to week 10 (week 52 and week 104) will be analyzed. The null hypothesis is that the average difference is zero. The alternative hypothesis is that the average difference has a nonzero value. Based on the results of a previous study [15], we aim to demonstrate a difference of 7 points or more with an α of .05, assuming an SD of 12, and assuming that each pair of measurements on the same individual correlates with approximately 0.5. With a sample size of 250 subjects, a power of >90% is achieved to detect the above mean of paired differences with a 2-tailed paired *t* test [16].

#### Exploratory Data Analysis

As an exploratory data analysis, correlations between the pMayo and exercise data and psychomotor data (such as sleep quality, stress, and fatigue) will be calculated.

### Definition of the Study Population

This prospective observational investigator-initiated trial will be conducted with 250 participants at approximately 50 sites all over Germany. The gender ratio will be balanced.

### Selection of Study Sites

Sites are IBD-experienced hospitals or gastroenterology practices in Germany. Site selection will be made by the study investigators. By signing the investigator agreement, each site confirms its fulfillment of all formal requirements for inclusion in the study and guarantees its compliance with data privacy laws and any other regulations about the execution of this observational study.

### Participant Criteria

The inclusion criteria for the FilgoColitis study include (1) the confirmed diagnosis of UC (according to the current guidelines from the German Society for Gastroenterology, Digestive and Metabolic Diseases and European Crohn’s and Colitis Organization), (2) the initiation of therapy with filgotinib at the physician’s discretion regardless of potential study participation, (3) age of the patient between 18 and 80 years at inclusion, and (4) in-label use according to filgotinib product information. The exclusion criteria include (1) planned surgical intervention with hospitalization, (2) history of malignant disease (except “nonmelanoma skin cancer”), and (3) contraindications for treatment with filgotinib (according to the product information).

### Study-Specific Examinations: The Use of Wearables

Within the scope of the additional examination, the 2 accelerometry-based wearables (SENS motion accelerometry leg sensor, and GARMIN smartwatch) will be provided by the clinical research organization. Participation in the additional examination is voluntary.

The wearables aim to improve data collection of physical activity behavior. Activity and psychomotor data are recorded by the provided trackers and transmitted to the server via the MyTARGET app.

**Figure 1:**
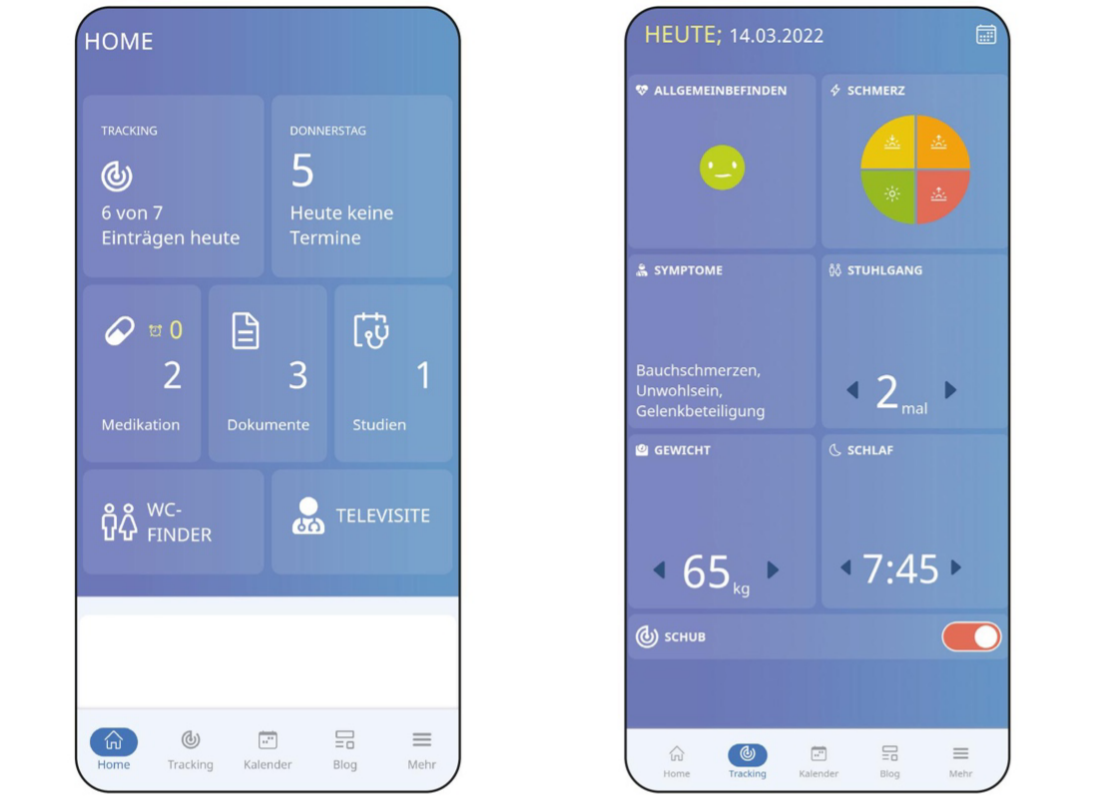
Homescreen and tracking area of the MyTARGET app.

The MyTARGET app (Figure 1) is a therapy support app for people with IBD. It was developed by the competence network IBD (Kompetenznetz Darmerkrankungen) and the German Association of Gastroenterologists (Berufsverband Niedergelassener Gastroenterologen Deutschlands) in close cooperation with experienced gastroenterologists, physician assistants, and patients to provide the best possible support for patients in their daily lives. The app is freely accessible and can be used by anyone. Within the app, there is a specific FilgoColitis study area, where only study participants have access via a QR code. In this area, patients can connect their wearables to their smartphones via Bluetooth. Once the devices are connected, a secure data transfer of the movement data to the study servers can take place assisted by the MyTARGET app. All data are stored only on the study server and are not passed on to the GARMIN company. The patients also receive a small output of their movement data.

The GARMIN watch may be worn for the entire study period of 24 months (day and night). If the physician and patient agree, it is desirable to be worn during the 10-week induction phase (day and night) or at least in the following 7 days after the baseline visit. For the follow-up visits, the GARMIN watch, if used, may be worn for 7 days after each follow-up visit (day and night).

If desired by the patient, the SENS motion sensor may be worn from the baseline visit and at each follow-up visit for 7 days (day and night). Both wearables may also be worn 3 days before the start of filgotinib therapy (baseline visit), if applicable. These movement data are not made available to the treating physician in a direct temporal context so as not to influence therapeutic decisions.

### Schedule of Visits

Visits at the study site are scheduled at inclusion (baseline visit week 0) and during follow-up (week 10, month 6, 12, 18, and 24). Follow-up visits are not fixed scheduled visits but are to occur when the study participant sees his or her physician as part of routine treatment.

### Documentation

Data are recorded using case report forms at the study sites and are transferred to an electronic case report form at the site. Data outlined in Table 1 are recorded during the baseline examination and at follow-up. In addition, each (serious) adverse event is recorded.

**Figure 2:**
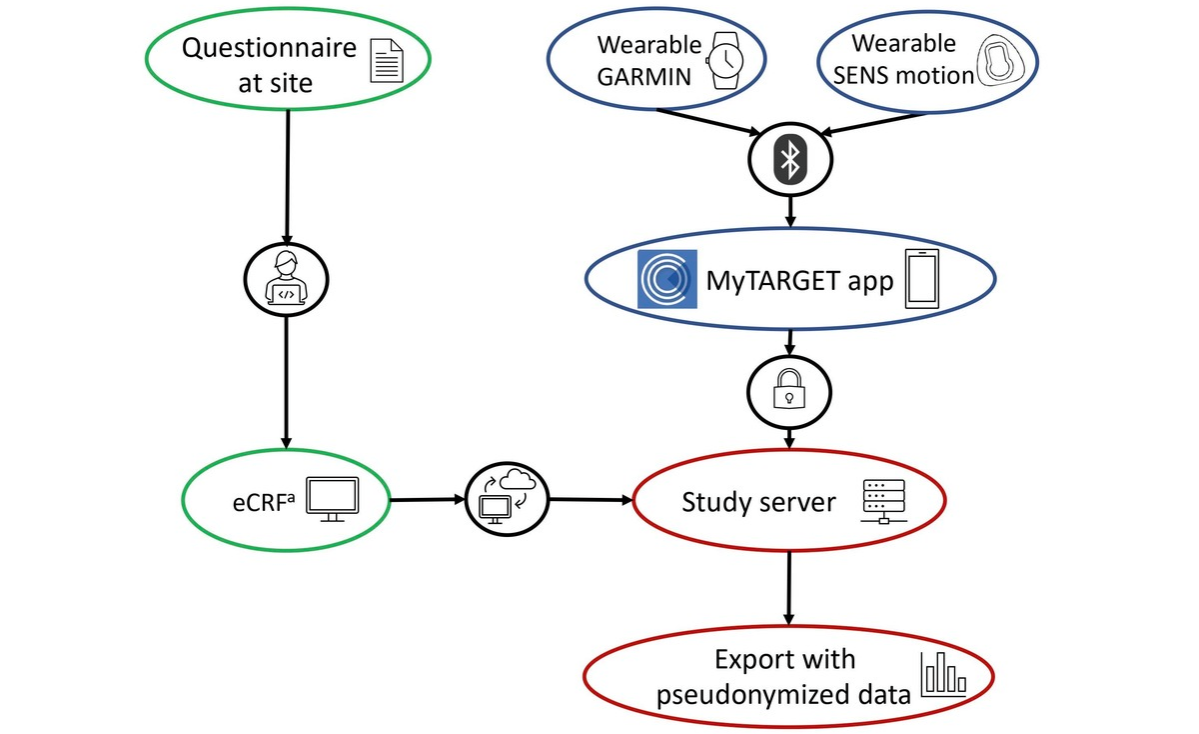
Transfer of the movement data from wearables to the study server. eCRF: electronic case report form.

The movement data of the additional wearable examination are recorded and stored locally as soon as the patient puts them on (Textboxes 1 and 2). Data are transferred from the wearables to the MyTARGET app on the patient’s smartphone via Bluetooth. The patient is given access to the study area via an individual QR code within the app. In this QR code, a digital pseudonym is stored, which enables the assignment of the movement data to the patient’s own study number. When the wearables and app are connected via Bluetooth, the data are automatically transferred from the devices to the MyTARGET app on the study participant’s cell phone and from there to the study server (Figure 2). There, the movement data are merged with the site-based collected data on the patient’s pseudonym.

**Table 1:** Clinical data collected in the FilgoColitis study stratified by baseline and follow-up visits.

Variable			Baseline visit	Follow-up visits (week 10, month 6, 12, 18, and 24)
**General information**				
	Date of patient visit		✓	✓
	FilgoColitis study number		✓	
	Diagnosis of ulcerative colitis		✓	✓
	**Study participation with wearables**		✓	✓
		GARMIN Fitness watch	✓	✓
		SENS motion ID	✓	✓
	Short pseudonym		✓	✓
**Demographic data**				
	Gender		✓	
	Date of birth		✓	✓
	Height		✓	
	Weight		✓	✓
	Waist circumference		✓	✓
	Smoking status		✓	✓
	Zip code		✓	✓
	Ethnic origin		✓	
	Social status		✓	✓
	Highest school qualification		✓	✓
	Member in the self-help organization German Crohn’s Disease and Ulcerative Colitis Association		✓	✓
	Sports activity		✓	✓
**Anamnesis**				
	Date of initial diagnosis (IBD^a^)		✓	
	First IBD symptoms (retrospective)		✓	
	Relatives with IBD		✓	
	Hospitalization		✓	✓
	Stool frequency previous night		✓	✓
	Start of filgotinib therapy (induction phase)		✓	
	Proctocolectomy		✓	✓
	Cancer diagnosis		✓	✓
	Fistula		✓	✓
	Stenosis		✓	✓
	Comorbidities		✓	✓
	Drug intolerances		✓	✓
**Current findings**				
	Montreal classification		✓	✓
	Current illness situation		✓	✓
	Laboratory parameters (C-reactive protein, fecal calprotectin, and hemoglobin)		✓	✓
	Endoscopy		✓	✓
	Extraintestinal manifestation		✓	✓
	Current medication		✓	✓
	Partial Mayo Score		✓	✓
**PROs^b^**				
	General quality of life (EQ-5D-5L)		✓	✓
	Disease-specific quality of life (sIBDQ^c^)		✓	✓
	Fatigue Questionnaire (IBD-F^d^)		✓	✓

^a^IBD: inflammatory bowel disease.

^b^PRO: patient-reported outcome.

^c^sIBDQ: Short Inflammatory Bowel Disease Questionnaire.

^d^IBD-F: Inflammatory Bowel Disease-Fatigue.

**Collected data from the wearable GARMIN vívosmart 4 within the additional examination.**

- Steps
- Heart rate
- Stress
  Recovery
  Low stress
  Medium stress
  High stress
- Sleep duration
- Sleep level
  Light sleep
  Deep sleep
  Rapid eye movement (REM) sleep

**Collected data from the wearable SENS motion within the additional examination. Duration and steps were collected for each movement type.**

- Lying or sitting rest
- Lying or sitting movement
- Standing
- Sporadic walking
- Walking
- Moderate intensity
- High intensity
- Cycling

### Patient Identification

All patient data are pseudonymized. Each patient will be identified by a patient identification number assigned at each study site. The investigator will keep a patient identification list, documenting the patient identification number with the patient’s full name, date of birth, sex, and date of informed consent.

The patient identification list is part of the investigator’s file and will remain at the site. The patient identification number comprises a 3-digit clinic number and a consecutive 3-digit number of recruited patients per study site.

### Study

#### Start of Patient Participation

Any patient who meets the inclusion and exclusion criteria of the study is a potential candidate. All potential candidates who come to the attention of the investigator will be consecutively informed of the opportunity to participate in the study. Written informed consent will be obtained before the commencement of documentation.

#### End of Patient Participation

The observation of each patient ends according to the schedule with the last study visit (month 24), after 2 years. A patient’s participation in the study will be terminated prematurely if at least one of the following criteria is met: (1) withdrawal of informed consent or death of the patient, (2) change of diagnosis, (3) lack of medical justification for further participation in the study, (4) premature termination of the complete study, or (5) subsequent discovery that not all inclusion criteria are met or that any exclusion criteria are met at baseline. A change in therapy does not result in study dropout. Furthermore, patients may be declared as lost-to-follow-up if they are no longer available and thus could not be documented at more than 2 consecutive visits.

#### Study Duration

This prospective study is planned for over 3.5 years (including 18 months of recruitment and 24 months of follow-up documentation per patient). The complete study is considered to have ended after all queries from the study coordination center have been answered by each study site, at the latest, 2 months after the last visit of the last patient.

#### Data Quality, Quality Control, and Assurance

The input fields in the web-based documentation form are stored with an input logic to avoid incorrect entries. In addition, remote monitoring of the data is permanently carried out and, if necessary, corresponding queries are made. Approximately 30% (n=75) of documented cases will be reviewed via in-house or on-site monitoring. Within the on-site monitoring, all study-relevant documents will be inspected and checked for completeness and current validity. In case of missing or implausible data, queries are generated and forwarded to the corresponding study site. This process is described in more detail in the monitoring manual.

#### Data Collection

Data collection of questionnaires (clinical data) and wearables data will take place on secured web servers based in Germany. The data will be transmitted and evaluated pseudonymously.

### Ethics Approval and Regulatory Aspects

The principal investigators and CED Service GmbH in Kiel are responsible for obtaining the required ethics committee approval as well as compliance with data protection regulations. Approval is obtained from the ethics committee of the medical faculty of the University of Kiel (D 609/21). The German Federal Institute for Drugs and Medical Devices (Bundesinstitut für Arzneimittel und Medizinprodukte), the “Kassenärztliche Bundesvereinigung,” the “Spitzenverbände der Krankenkassen,” and the “Verband der Privaten Krankenversicherungen e.V.” have been notified of this noninterventional study according to §67 of the German Medicinal Products Act (Arzneimittelgesetz). A final report will be sent to the responsible federal authority after completion of the data collection according to §67 (6) of the German Medicinal Products Act. Furthermore, the study is published in the public German Clinical Trials Register (DRKS00027327).

Before enrollment in the study, the investigator will inform each patient of the nature, significance, risks, and scope of the study and the patient’s right to withdraw from the study at any time without prejudice. The patient will be given an informed consent form describing the study in nonscientific and generally understandable language. Each patient must consent in writing to participate in the study. The patient must be given sufficient time to decide and ask questions before signing the consent form.

## Results

The enrollment commenced in December 2021 and was still open at the date of submission. The study is expected to complete in June 2026. After 6 months of study initiation, 69 patients were enrolled in FilgoColitis. The median age of patients is currently 41.7 (IQR 28.7-56.0) years; 52% (n=36) of patients were male and had a disease duration of 7.4 (IQR 4.3-14.8) years. In total, 90% (n=62) and 23% (n=16) of patients were pretreated with biologics and small molecules, respectively. Wearables were accepted by 80% (n=55) of patients.

## Discussion

### The Rationale for the Study

Treatment of moderate to severe UC remains challenging with approximately 30% of patients benefiting from biologics. Further modes of action are therefore needed. JAK inhibitors play a role by inhibiting the Janus kinase/signal transducers and activators of transcription pathway, thereby slowing down the inflammatory process [17].

The first to market in 2019 was a pan JAK inhibitor, tofacitinib. It is hoped to achieve a more specific effect by inhibiting individual JAKs in a more targeted manner: filgotinib is a JAK1 preferential inhibitor, and therefore fewer side effects are expected.

The pivotal SELECTION study [18] showed significantly higher efficacy (clinical remission) in patients receiving 200 mg filgotinib therapy when compared to a placebo in both the induction and maintenance phases. The incidence of serious adverse events was not higher in the filgotinib groups compared to the placebo group.

The safety profile and the effectiveness of objective disease scores as well as PROs under real-world conditions have not yet been studied. Therefore, the FilgoColitis study was initiated shortly after approval in Germany and Europe. The study focuses on PROs, which will be collected both subjectively via questionnaires but also using wearables to provide objective data on activity behavior and psychomotricity. In addition to abdominal pain, fatigue is reported by many patients as a common complication [12]. Therefore, the IBD-F is applied, for which the German version has recently been validated [14]. The scale of the IBD-F questionnaire was developed to be used in the diagnosis of fatigue or to monitor fatigue levels over time. The IBD-F scale consists of 3 sections: section I identifies the level and duration of fatigue, section II assesses the impact of fatigue on daily activities, and section III identifies causes and other factors related to fatigue. The FilgoColitis study will analyze if there is a correlation between the objective movement measures, disease activity, and fatigue.

### Justification for Study Design

The study is conducted nationwide and multicentrally with specially selected sites to achieve higher representativeness of the study results for the entire population. Since the study’s primary objective is to observe the effects of filgotinib therapy on QoL and psychomotor markers, a single-arm study design is warranted. Furthermore, the study aims to investigate to what extent it is possible to have an additional observational outcome tool for monitoring disease activity in patients with IBD by using wearables.

### Wearables

Since exercise and sleep are associated with QoL, 2 wearables are used in this study to enable measurement of the patients’ movement and sleep comprehensively. We hypothesize that early changes in wearable parameters (eg, increase of step counts and normalization of heart rate variability) are potentially associated with long-term improvement of QoL. Other studies show similar results. van Langenberg et al [19] reported significantly poorer sleep quality and less physical activity in patients with Crohn disease compared with healthy control subjects, as measured by an accelerometer over 7 days. Similarly, Wiestler et al [6] found significant associations between physical activity and the disease-specific QoL and disease activity in patients with IBD.

In the FilgoColitis study, 2 wearables are used. The leg accelerometry sensor SENS motion is a medical device, whereas the GARMIN smartwatch vívosmart 4 is a simple fitness watch. We aim to show that nonmedical devices can also be used in clinical studies to measure physical activity and can be related to other important outcomes. The advantages of nonmedical devices are higher affordability and easier usability. Thus, we hope to be able to scientifically demonstrate in the FilgoColitis study that less expensive, widely used devices are also suitable for the monitoring of patients with UC. Nevertheless, it must be noted that nonmedical devices can also be less valid and accurate. In an unpublished field study prior to commencement of the FilgoColitis study, we successfully showed the comparability of both wearables in an on-site test (data not shown).

To achieve meaningful results from the additional examination, the researchers are dependent on a high level of compliance from the study participants. For the success of the study, patients who voluntarily participate in the additional examination must wear their wearables at the desired times for the desired duration. However, since the FilgoColitis study is noninterventional and due to its voluntary nature in the additional examination, decreasing compliance of study participants must be expected. Because of the longer study duration, patients may forget to put the wearables back on for the visit or to transfer their data. Willingness to wear a watch or leg sensor visibly on the body may also drop over the study course. These assumptions were made based on findings from researchers in other digital studies where a decline in compliance over the course of the study was observed. One review showed that compliance is usually very high at the start of the study and then declines rapidly over the first weeks and months. However, success factors such as user training, health education, and maintaining strong participant motivation were also identified to positively influence compliance [20].

The compliance of the study participants cannot be enforced, but the framework conditions can be designed in the best possible way to achieve the highest possible compliance. Therefore, measures were also introduced in the FilgoColitis study, such as the creation of detailed instructions and web-based tutorials or active technical support by the study team. If the study size is smaller than expected due to low compliance, statistical recalculations must be performed to ensure that the results are still statistically meaningful. Nevertheless, if compliance within additional examination is so low that no meaningful results can be obtained, as a consequence the results can only be examined in relation to the primary and secondary outcome measures. However, due to the measures mentioned above, we hope to achieve the highest possible compliance of the study participants.

The use of new technologies such as smartwatches or leg sensors has many advantages in studies. Examples are higher recruitment rates, faster feasibility of the study, and the collection of a broader range of health data [21,22]. Atreja et al [23] summarize in the review on remote monitoring of patients with IBD that in the near future this rapidly evolving technology will become a mainstream tool in standard patient care practices. Nevertheless, certain obstacles must also be expected. Technical problems may occur at any time, which means that important participant movement data cannot be recorded or transmitted and is therefore lost. In addition, the additional examination is not feasible for everyone. Patients without their own smartphones cannot participate, and certain prior knowledge of smartphone use is also assumed. Due to the partly complicated procedure, older people may be excluded due to excessive demands or lack of knowledge. However, studies show that older people are generally positive about smartwatch technologies for measuring PROs. According to Manini et al [24], the aspect of user-friendliness is one of the most important points that must be given special consideration during design. Therefore, elements such as detailed explanations and descriptions, big visible buttons, and a frequently asked question area were added to the MyTARGET study app. The goal was to create the greatest possible user-friendliness, so that older study participants are also attracted to the additional examination within the FilgoColitis study.

### Strengths and Limitations

The FilgoColitis study is the first large real-world study of filgotinib. One strength is the prospective design of the study. In addition, the primary outcome parameter is a PRO, namely QoL. In particular, the assessment of fatigue represents a modern approach in IBD research. Finally, the use of new technologies that objectively measure exercise, sleep, and stress behavior makes the study unique.

The study also has some limitations: a selection bias cannot be excluded in the FilgoColitis study and should also be considered in future analyses. To recruit a broad representative population of study participants and overcome the selection bias, we distributed the study sites all over Germany and included patients with a large age range (18-80 years). In addition, physicians consecutively enrolled eligible patients in the study. Moreover, filgotinib is the first oral preferential JAK1 inhibitor, and its approval has also opened up new strategies for the treatment of patients with UC. Due to the newly emerging treatment opportunities, it can be assumed that mainly last liners, meaning patients who have already taken several biologics without success, will receive the new drug and will be included in the study. Accordingly, it can be assumed that these patients tended to be in poor health prior to study inclusion and have a low QoL. However, the effect that filgotinib may have on QoL can still be well examined by surveying QoL at baseline. Thus, individual improvement or worsening of QoL over the course of the study can be evaluated for all participants without bias regarding the prevailing health status. The selection bias is tried to be avoided accordingly.

In addition, the FilgoColitis study is conducted using written questionnaires completed by the patients and physicians. On the one hand, we do not see limitations with regard to the physicians, since the remote monitoring system allows queries to be asked at any time regarding open or unclear answers. On the other hand, however, there could be omitted questions or illogical answers with regard to the patients, which could negatively influence the analysis of the study objective. To counteract this limitation, however, the attending physicians and the trained study nurses work closely with the patient so that questions can be asked at any time in the event of ambiguities or problems. In this way, as many missings as possible are to be avoided.

Nevertheless, with our efforts to eliminate the mentioned limitations, we aim to use the observational FilgoColitis study to draw significant correlations between the QoL and psychosocial well-being of patients with UC on filgotinib therapy over the long-term course under real-world conditions. With the results of the study, we intent to assist physicians in making future treatment decisions regarding the prescription of filgotinib.

### Conclusions

In summary, in the FilgoColitis study, we aim to find out how the new JAK1 inhibitor filgotinib affects the QoL of patients with UC in clinical practice with real-world conditions and how safe the therapy is. By using wearables, we assume to obtain superior data to assess patients’ QoL. We also hope to prove that the use of wearables could be an additional observational outcome tool to monitor disease activity in patients with IBD. With the multicenter approach, large study population, and long study duration, we aim to obtain meaningful and valid data on the safety and effects of filgotinib therapy.

## Abbreviations

EQ-VAS: EQ visual analog scale
IBD: inflammatory bowel disease
IBD-F: Inflammatory Bowel Disease-Fatigue
JAK: Janus kinase
PRO: patient-reported outcome
QoL: quality of life
sIBDQ: Short Inflammatory Bowel Disease Questionnaire
UC: ulcerative colitis

## Data Availability

The data sets generated and analyzed during this study are available from the corresponding author upon reasonable request.
